# Latent space reconstruction for missing data problems in CT

**DOI:** 10.1002/mp.17910

**Published:** 2025-06-04

**Authors:** Anton Kabelac, Elias Eulig, Joscha Maier, Maximilian Hammermann, Michael Knaup, Marc Kachelrieß

**Affiliations:** ^1^ Division of X‐Ray Imaging and Computed Tomography German Cancer Research Center (DKFZ) Heidelberg Germany; ^2^ Faculty of Physics and Astronomy Heidelberg University Heidelberg Germany; ^3^ Medical Faculty Heidelberg Heidelberg University Heidelberg Germany

**Keywords:** computed tomography, deep learning, missing data

## Abstract

**Background:**

The reconstruction of a computed tomography (CT) image can be compromised by artifacts, which, in many cases, reduce the diagnostic value of the image. These artifacts often result from missing or corrupt regions in the projection data, for example, by truncation, metal, or limited angle acquisitions.

**Purpose:**

In this work, we introduce a novel deep learning‐based framework, latent space reconstruction (LSR), which enables correction of various types of artifacts arising from missing or corrupted data.

**Methods:**

First, we train a generative neural network on uncorrupted CT images. After training, we iteratively search for the point in the latent space of this network that best matches the compromised projection data we measured. Once an optimal point is found, forward‐projection of the generated CT image can be used to inpaint the corrupted or incomplete regions of the measured raw data.

**Results:**

We used LSR to correct for truncation and metal artifacts. For the truncation artifact correction, images corrected by LSR show effective artifact suppression within the field of measurement (FOM), alongside a substantial high‐quality extension of the FOM compared to other methods. For the metal artifact correction, images corrected by LSR demonstrate effective artifact reduction, providing a clearer view of the surrounding tissues and anatomical details.

**Conclusions:**

The results indicate that LSR is effective in correcting metal and truncation artifacts. Furthermore, the versatility of LSR allows its application to various other types of artifacts resulting from missing or corrupt data.

## 1 INTRODUCTION

Computed tomography (CT) images are typically reconstructed using algorithms of filtered backprojection (FBP) type, of iterative reconstruction type, or with the help of deep learning. The goal is to reconstruct an unknown image $f\in R^{n}$ given observed measurements $p\in R^{m}$:

(1) Rf=p+η,

where $R\left(\cdot \right):R^{n}\to R^{m}$ represents the Radon transform, that is, the forward projection and $\eta \in R^{m}$ is additive noise. A high quality CT image reconstruction relies on this underlying inverse problem to be well‐posed in order to reconstruct artifact‐free images. However, in some clinical situations this is not the case, that is, m≤n, rendering the problem ill‐posed. This can lead to the rise of artifacts, and may make a medical diagnosis based on CT images difficult and sometimes impossible. Two clinical examples for missing or corrupt data problems are lateral truncation and metal artifacts.

### 1.1 Solving inverse problems with generative models via data consistency

In this work we introduce a novel method, latent space reconstruction (LSR), for estimating data in CT in missing or corrupt data settings. LSR includes an iterative optimization of latent data representation, while enforcing data consistency with measurements. Recent advancements in generative models have significantly influenced the development of methods for solving such ill‐posed inverse problems. These methods often leverage learned priors to guide reconstruction, enforce data consistency, and recover missing information.

For compressed sensing of natural images, Bora et al.,^1^ find an image that minimizes the measurement error ||AG(z)−p||, with G being a generative model and A the Radon transform, by optimizing the latent space variable z directly. However, they perform experiments on low dimensional (64×64) datasets and do not consider measurements from medical imaging.

Similarly, Yeh et al.^2^ propose a semantic inpainting framework based on a generative adversarial network (GAN) to reconstruct missing image regions by optimizing a context loss, which enforces consistency with visible data, and a prior loss, which ensures the generated images remain realistic. However, their method is not designed for domain‐specific measurement models, such as those in CT imaging.

Shah and Hegde^3^ propose using a GAN as a prior to solve inverse problems like compressed sensing. Their method employs a projected gradient descent algorithm to iteratively refine solutions, ensuring they lie on the manifold of GAN‐generated natural images. Unlike LSR, which uses a variational autoencoder (VAE) to specifically model uncorrupted CT images, this method focuses on natural image distributions and lacks explicit consistency in the rawdata domain.

In the medical imaging domain, Tivnan and Stayman^4^ introduce the MRoD algorithm, which combines a manifold‐based data‐driven prior with a physics‐based statistical model to reconstruct CT images. It estimates a “manifold component” that captures common features among all patients and a “difference component” that fits measured data, allowing it to reconstruct rare features not present in the training data. While the algorithm incorporates a data‐consistency term to guide the difference component, the measured data p itself is not directly preserved in the final output, as the reconstruction is a combination of generated components. In contrast, LSR explicitly retains all the information contained in p and only extrapolates into regions with missing or corrupted data, ensuring data consistency of the final reconstruction. Furthermore, while the experiments in ref. [4] are limited to simulations of digital phantoms, we demonstrate the practical applicability of LSR on a variety of patient measurements from different CT systems.

Song et al.^5^ introduce ReSample, an algorithm leveraging Latent Diffusion Models to solve inverse problems with hard data consistency. ReSample alternates between optimizing latent variables and remapping them onto the noisy data manifold. However, its reliance on iterative stochastic resampling makes it computationally expensive. While LSR also optimizes with a comparable hard data consistency, it explicitly retains all the information contained in p and only extrapolates into regions with missing or corrupted data.

### 1.2 Missing data: Truncation artifacts in CT

Lateral truncation is a missing data problem in CT imaging, where parts of the patient laterally exceed the field of measurement (FOM). The lateral FOM is the region within the x‐y‐plane where each voxel is viewed under 180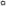 or more. In clinical practice, truncation most often occurs with patients that are not centered properly on the table, obese patients, or when using C‐arm systems, which generally are equipped with small, say, 30to40 cm, flat detectors and thus feature small FOMs of about 15to20 cm in diameter. Truncation causes cupping‐like artifacts inside the FOM, which become apparent especially at the FOM edges. Furthermore, regions outside the FOM cannot be reconstructed at all since they are seen by less than 180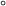 and thus suffer from severe limited angle artifacts.

Over the last decades, a number of methods for detruncation, that is, truncation artifact correction and field of view (FOV) extension have been proposed. Techniques for the reconstruction of truncated projection data can be categorized into three types. The first group of techniques are data completion methods, with many methods estimating the missing data through various extrapolation techniques. Hsieh et al.^6^ used the size and slope of a water cylinder fitted into each projection to estimate a suitable projection extension. Other extrapolation methods include elliptical extrapolation combined with consistency conditions, to estimate a convex hull of the patient,^7^ symmetric mirroring of the neighboring projection data at the edge of the FOM^8^ or hybrid extrapolation algorithms.^9^

A more sophisticated extrapolation uses information from a patient atlas, where the atlas patients are registered to the current patient and then forward projected.^10^

The second group consists of methods based on iterative reconstruction. Iterative reconstruction often involves creating an objective function that incorporates observed data and prior knowledge (e.g., about the object to be reconstructed or parameters of the CT system), and then deriving the solution by optimizing this function.^11^, ^12^ Recent advancements include hybrid techniques, such as those introduced in ref. [13] which incorporate region‐based constraints combining total variation and $\ell_{1}$‐norm regularization to suppress truncation artifacts.

DL‐based methods are a third group of algorithms. DL‐based detruncation methods proposed in the literature can be categorized based on their data utilization: some methods work directly with measured projection data (sinogram‐based methods), while others operate on the reconstructed CT images. Rawdata‐based networks^14^, ^15^ act directly on the incomplete (truncated) measured projection data. These are typically extended with a convolutional neural network, like a U‐Net^16^ and the extended sinogram is reconstructed. For image‐based methods, such as refs. [17, 18], the task of FOV extension can be compared to semantic image outpainting methods on natural images.^2^ The truncated area is usually defined via a mask, and a network is trained to inpaint the missing regions. Dual‐domain based methods^19^, ^20^ combine the previous two methods and use two deep neural networks, one in the projection data domain and one in the image domain.

### 1.3 Corrupted data: Metal artifacts in CT

One example of a corrupt data problem are metal artifacts. Metal components inside the body of a patient have a higher attenuation of polychromatic x‐ray beams than soft tissue or bones. This results in scatter, beam hardening, photon starvation, edge gradient effects and their combination, which in return can cause metal artifacts that typically include bright and dark streaks.

Most proposed methods for metal artifact reduction (MAR) fall in one of the following categories: sinogram inpainting methods, iterative methods or DL‐based methods. The sinogram inpainting techniques usually employ interpolation of the metal trace in the sinogram, such as linear interpolation,^21^ spline interpolation^22^ and wavelet interpolation^23^. Such approaches might produce nonsmooth interpolation boundaries, which can in turn cause notable secondary artifacts in the reconstructed CT images. To mitigate this problem, the normalized metal artifact reduction (NMAR)^24^ method was proposed based on linear interpolation under the constraints of prior images to reduce metal artifacts.

Iterative MAR generally refers to the process of iteratively reconstructing CT images to decrease metal artifacts. Most approaches are grounded in the algebraic reconstruction technique (ART)^25^ or employ statistical iterative^26^, ^27^ reconstruction techniques.

In recent years, DL‐based methods have shown great potential at reducing metal artifacts. DL‐based MAR methods can work in the sinogram domain,^28^ image domain,^29^, ^30^, ^31^ or both.^32^, ^33^ The majority of deep learning‐based MAR methods are supervised, relying on paired metal artifact CT and clean CT data to train neural networks.

In this work we introduce a novel method, LSR, for estimating data in CT in missing or corrupt data settings. We evaluate the performance of LSR: first for truncation artifact reduction, and second for metal artifact reduction.

## 2 MATERIALS AND METHOD

### 2.1 LSR method

In the following, we will describe LSR for the 2D case. Note, that our framework can perform reconstruction of 3D data via slice‐wise application. CT images are denoted by f(x,y), with x and y being the spatial dimensions. Non‐corrupted raw projection data are denoted by p(α,β), with α being the projection angle and β being the angle of a ray within the fan. Corrupted rawdata, where some data are missing, invalid, or cannot be used, are denoted by q(α,β). X is representing the x‐ray transform, that is, the forward projection. In our study, the forward projection X computes line integrals over the reconstructed images. This same X is used for generating the ground truth sinogram p(α,β) and the corrupted sinogram q(α,β). We denote with $X^{-1}$ the FBP, that is, the inverse of the forward projection.

First, a neural network is trained to learn a complete representation of uncorrupted CT images, which do not exhibit missing data in the projection domain. This neural network must have a regularized latent space that enables generative modeling. In this study, we employed aVAE^34^, consisting of an encoder E and a decoder D. Future work could consider other types of generative models such as GANs^35^ or diffusion models. A VAE fulfills the condition, because its latent space is designed to resemble a parameterized probability distribution, enabling balanced sampling, and its decoder can generate new data by reconstructing images from points sampled within this latent space. Balanced sampling is important for LSR because it ensures that close points in the latent space correspond to similar generated axial slices, maintaining consistency and interpretability. Unlike a regular autoencoder, where the latent space may not support smooth interpolation or meaningful sampling, a VAE enforces a continuous and structured latent space.

After training, the encoder is no longer used. A latent vector z can be sampled from the latent space, which the decoder of our model can now map to a CT image D(z). This generated image is then forward projected to obtain a sinogram XD(z). In order to reconstruct an image from incomplete measurement data, LSR iteratively searches for the point z in latent space, that best matches the incomplete measurement data in rawdata space. We find z by solving the following optimization problem

(2) $$z=\arg\min_{z}\;∥M\left(XD(z)\right)-q∥,$$

where $M\left(\alpha ,\beta \right)\in {\left\{0,1\right\}}^{n\times m}$ represents a task‐specific mask operator, being one for rays that have been measured and zero for non–measured or corrupt rays. n is the number of distinct projection angles, and m is the number of rays for each projection angle.

Once converged to an optimal point in the latent space z, the decoder of our model can now map this optimal point to a generated CT image that has maximum data consistency with the incomplete measurement data in the raw data domain. The measured incomplete rawdata q are then extrapolated in the missing regions with the values from XD(z). The LSR‐corrected sinogram is then

(3) $$p_{\text{corrected}}=M\cdot q+\left(1-M\right)\cdot XD\left(z\right).$$

To obtain the final corrected image, the reconstruction is performed using standard FBP:

(4) $$x_{\text{corrected}}=X^{-1}\;p_{\text{corrected}}.$$

For the truncation case study, we investigate truncation to a 150mm FOM to mimic typical C arm FOMs. Truncation simulation is achieved by the truncation mask $M_{15\;\mathrm{cm}}$, that is zero at an appropriate number of the outer left and right detector channels.

**FIGURE 1:**
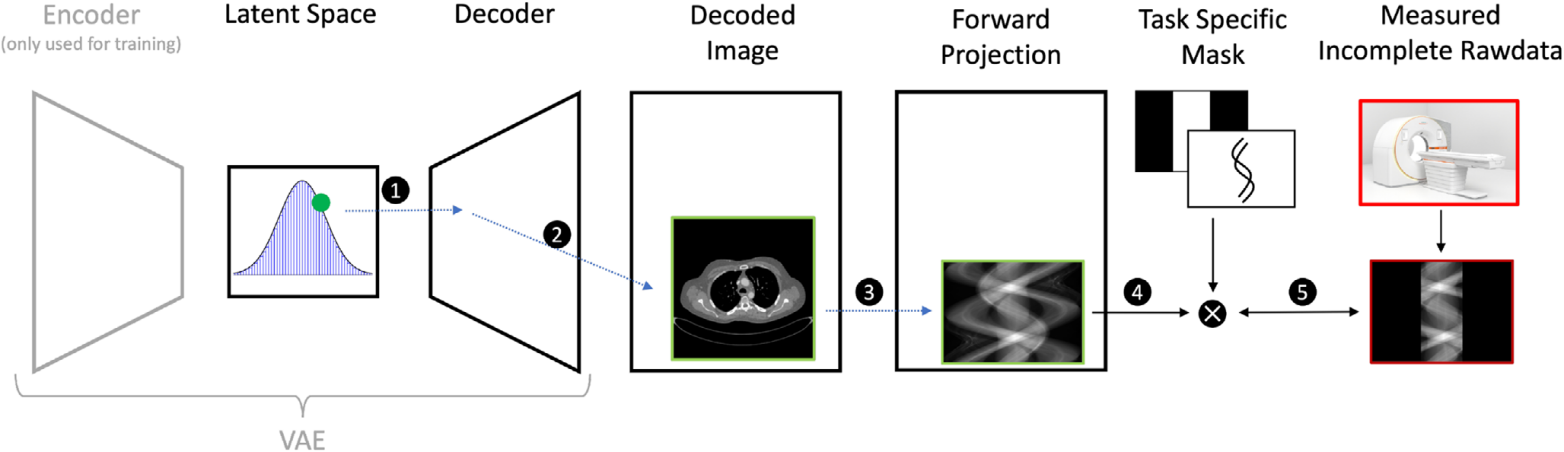
LSR optimization scheme: (1) Sampling a point z from the latent space. (2) Generating a non‐corrupted CT image D(z) from the sampled point. (3) Forward projecting the newly generated image into the rawdata domain. (4) Multiplying with a task specific mask. (5) Quantify agreement with the measured but incomplete rawdata and perform gradient descent. Repeat until the optimal z is found.

For the metal artifact case study, we segment the metal in the reconstructed CT scan using simple thresholding. Forward‐projecting the metal part produces a metal trace in the sinogram, which can be used to create a mask 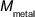 for the area in the sinogram that requires extrapolation, with 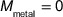 above the threshold value and 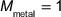 everywhere else (Figure 1).

### 2.2 LSR optimization

To solve the LSR minimization problem (2), we employed the gradient‐based Adam optimizer^36^ for the majority of optimization steps. For the final refinement, the quasi‐Newton limited‐memory Broyden–Fletcher–Goldfarb–Shanno (L‐BFGS)^37^ algorithm was utilized to ensure convergence to the local minimum.

**FIGURE 2:**
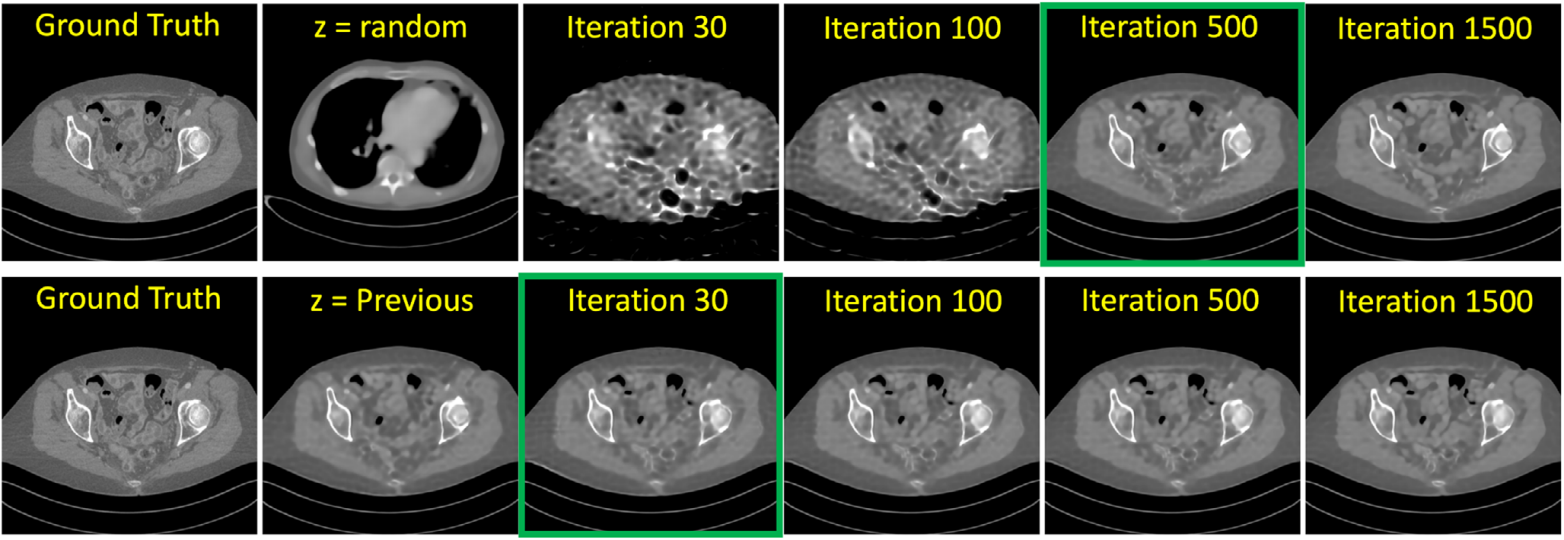
Original image and generated image D(z) for different numbers of optimization steps. Top row: starting from a randomly initialized latent space vector z∼N(0,1) with N(0,1) denoting a normal distribution of zero mean and unit variance. Bottom row: starting from the optimal latent space point obtained earlier along the axial direction. The green box indicates the number of sufficient steps for each case.

Figure 2 illustrates the reconstruction results for a varying number of optimization steps. We empirically found for most tasks, that, when starting from a random initialization, around 500 steps are required to achieve good results. Or, by utilizing the previous slice as a prior for initializing the latent variable, around 30 steps are required to achieve a sufficient outcome.

### 2.3 Network and data sets

Most deep learning‐based methods for addressing missing data in CT imaging use networks that are specifically tailored to their respective missing data problems.^14^, ^15^, ^18^, ^38^ In contrast, the network employed for LSR learns a latent representation of uncorrupted images, rendering the training process independent of the particular missing data completion task. This allows us to use the same network for all subsequent case studies. The exact architecture of our network is described in the Appendix.

Our network was trained on clinical CT data, acquired with a Siemens Somatom Force CT system (Siemens Healthineers, Forchheim, Germany). Our data set consists of 85 adult patients (60 male, 25 female), with a total of 94 117 slices. The images were part of a dual energy scan and are all acquired at 70kV. They are reconstructed into a 512×512 matrix with a slice thickness of 0.6mm and an axial voxel spacing between 0.69 and 0.98mm.

From these 85 patient data sets we generate complete sinograms p(α,β) for each of the 94 117 slices, by forward‐projecting in fan beam geometry with N=720 projections covering an angular range of 180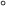 with M=500 detector elements. These sinograms serve as the ground truth rawdata. Note that these data include all physical effects present in the image data that were not accounted for during the reconstruction process, including noise and potentially scatter^1^. Limited‐data sinograms are then created by multiplying p(α,β) with a task‐specific mask M

(5) q(α,β)=M(α,β)·p(α,β).

Since images were reconstructed from that 70 kV data using a simple FBP, the sinograms p(α,β) and q(α,β) therefore correspond to data measured with a polychromatic 70 kV spectrum. For our LSR, the network does not predict energy‐dependent attenuation coefficients but was trained to predict the reconstructed images from the clinical CT dataset. Applying our method to data acquired with different spectra than that data would require either retraining the network (or separate networks) on data acquired with different spectra. This ensures that the network can generalize to such data. Alternatively, the network could predict energy‐dependent attenuation coefficients. At test time, a polychromatic forward‐projection can then be performed using a spectrum appropriate for the measurement at hand.

The patients were randomly split into 56 patients for training, 13 for validation and 13 for testing. An additional three patients had metal implants and were used to evaluate LSR for the task of metal artifact reduction.

Additionally, the LSR method was applied to rawdata from two cone‐beam CT (CBCT) measurements: one acquired with a Varian HyperSight system (Varian Medical Systems, Palo Alto, United States) at 125 kV tube current, and another from an onboard CBCT of a Varian ProBeam360 at 140 kV tube current.

## 3 RESULTS

### 3.1 Truncation artifacts in CT

**FIGURE 3:**
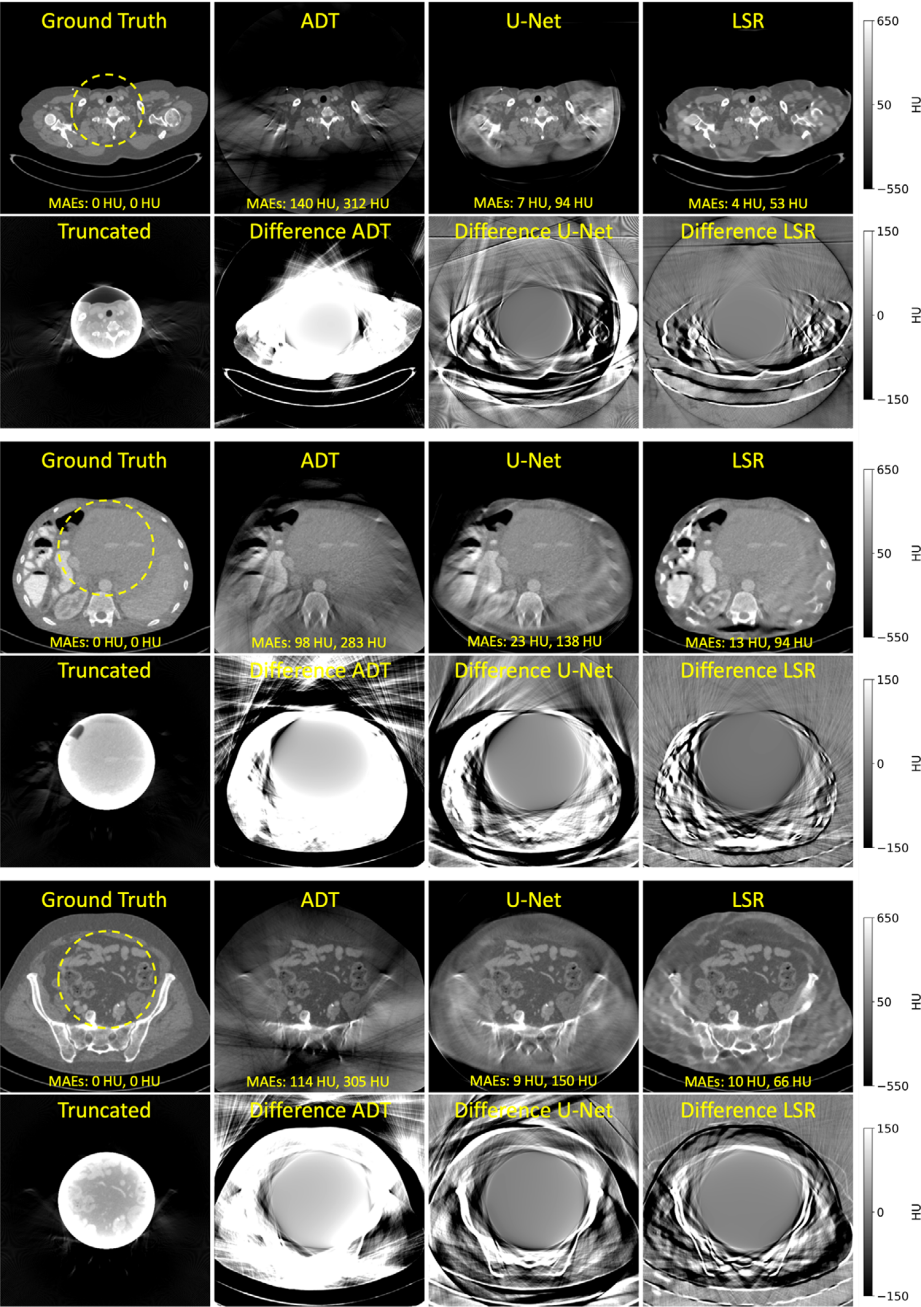
Detruncation from 15cm FOM to 50cm. Images C=50HU,W=1200HU. Difference images C=0HU,W=300HU. MAEs refer to the inner and outer regions, respectively.

Figure 3 shows the performance of LSR compared to a classical method^7^ (ADT) and a DL based method^15^ (U‐Net). For each sample we show the ground truth image, the classical detruncation result, the DL result and the LSR‐based detruncation as well as the difference images of each method to the ground truth. Quantitative results for the entire test data set can be seen in Table 1. Further experiments on LSR‐based detruncation in simulated scenarios with improperly centered or larger patients are provided in Appendix A.3.

**TABLE 1:** Quantitative comparisons of the LSR performance.

	Uncorrected	ADT^7^	U‐Net^15^	LSR
$\bar{\mathrm{MAE}}$ inside FOM $\left[\text{HU}\right]$	703.5 ± 178.2	112.6 ± 55.5	26.2 ± 24.0	15.0 ± 9.5
$\bar{\mathrm{MAE}}$ outside FOM $\left[\text{HU}\right]$	568.5 ± 147.9	313.2 ± 76.5	134.4 ± 40.7	102.6 ± 75.5
$\bar{\mathrm{MAE}}$ of sinogram	1.19 ± 0.39	0.91 ± 0.33	0.23 ± 0.17	0.20 ± 0.07

Abbreviations: FOM, field of measurement; LSR, latent space reconstruction; MAE, mean absolute error.

For evaluation, we use the average mean absolute error (MAE) to the ground truth inside the FOM, outside the FOM, and in the rawdata domain and the standard deviation of the individual MAE values from our N test data scans.

Regions outside the FOM have limited diagnostic value because their projection data are primarily generated by the VAE followed by forward projection, rather than directly measured. Consequently, the MAE outside the FOM is greater than the corresponding measure within the FOM. However, the MAE in these regions can still serve as an indicator of the accuracy of the data generated by the VAE. Furthermore, an extended FOM can be advantageous in specific applications, such as dose estimation scenarios.

**FIGURE 4:**
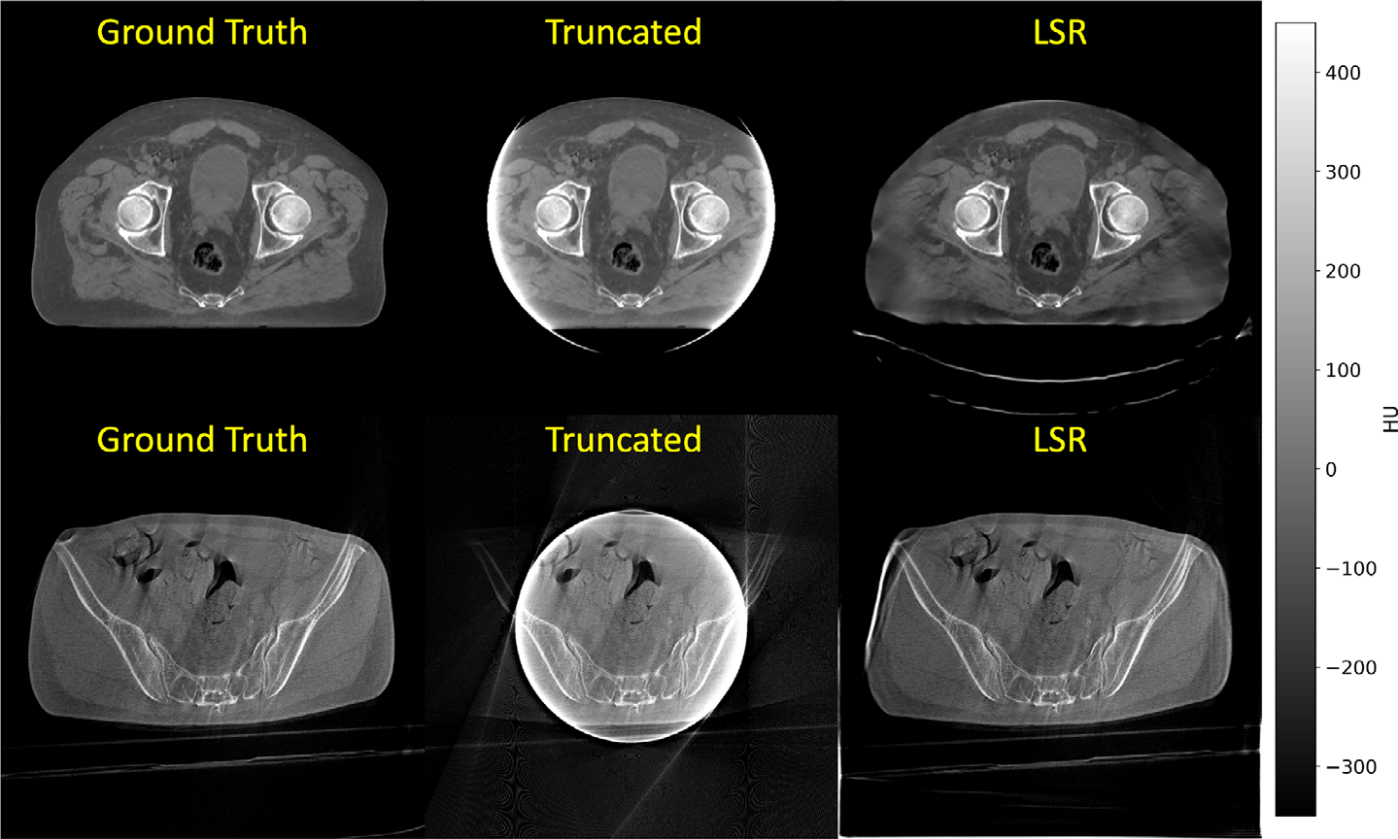
Detruncation of two CBCT measurements at a 20 cm FOV. Top row: Measurement from an onboard CBCT of a Varian ProBeam360 with a tube voltage of 140 kV. Bottom row: Measurement from HyperSight with a tube voltage of 125 kV. Window level: C=50 HU, W=800 HU.

Additionally, Figure 4 shows the performance of LSR on CBCT data without retraining the VAE. The results indicate that despite only having seen reconstructions from 70 kV measurements the network generalizes well to measurements that were taken at different tube voltages and contain varying levels of scatter and noise.

Overall, we observe that images corrected by LSR exhibit effective artifact suppression within the FOM, along with a substantially improved FOM extension compared to other methods.

### 3.2 Metal artifacts in CT

**FIGURE 5:**
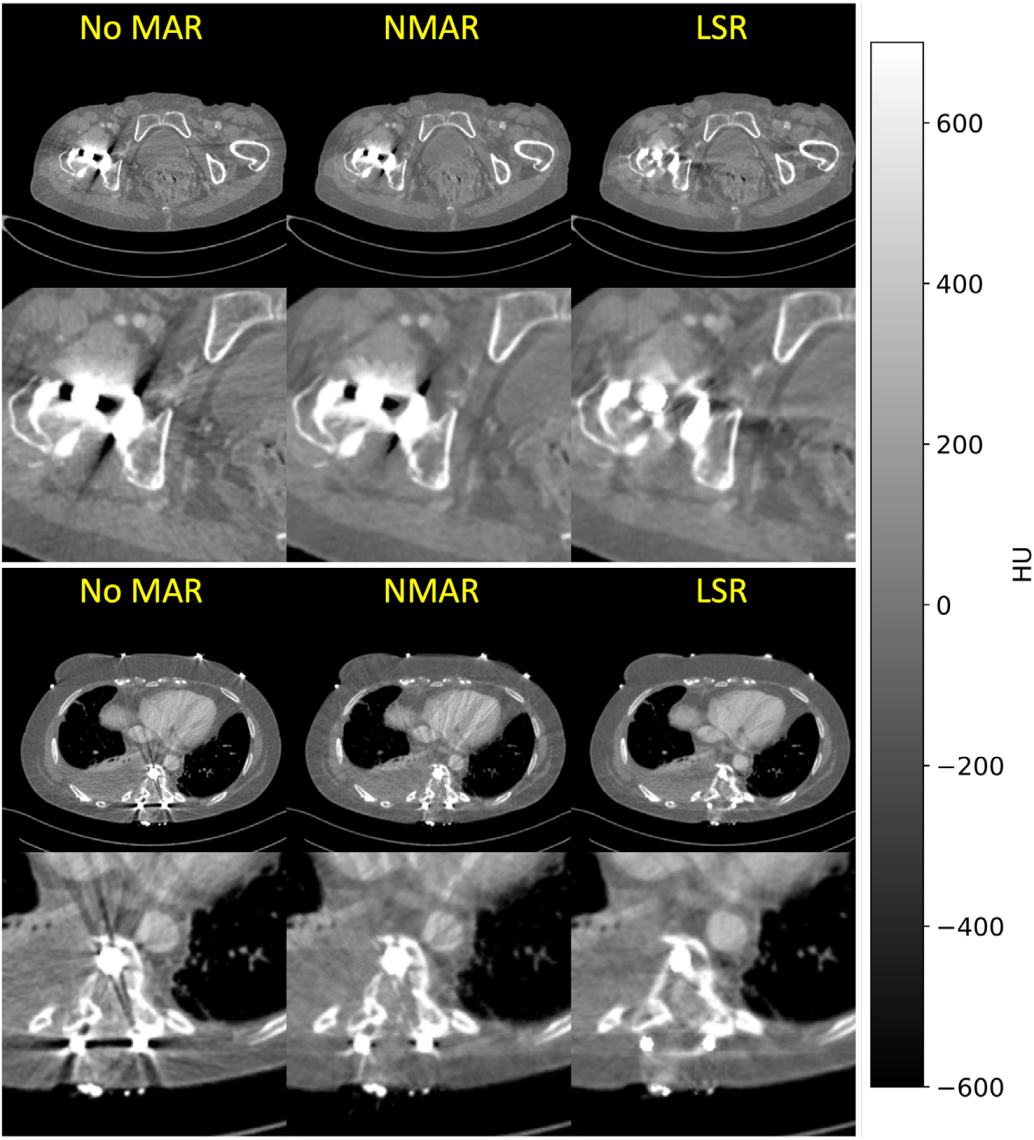
Qualitative results for LSR MAR, compared to NMAR. C=50HU,W=1300HU.

Figure 5 shows the performance of LSR compared to NMAR.^24^ Since there exists no artifact‐free ground truth for these patients we restrict ourselves to a qualitative evaluation. For both patients shown here, the No MAR image (left) is significantly impacted by streak artifacts, which obscure critical anatomical structures. The NMAR image (center) shows a reduction in these artifacts, though some remain, particularly close to the metal. The LSR image (right) demonstrates the most effective artifact reduction, providing a clearer view of the surrounding tissue and anatomical details.

However, it is also important to note that the LSR technique introduces the formation of streak artifacts tangent to the metal objects. These artifacts are introduced when the transition between original and interpolated projection data is not sufficiently smooth.

## 4 DISCUSSION

In this work, we introduced a novel deep learning‐based framework, LSR, to address missing and corrupt data challenges in CT image reconstruction. For the implementation of LSR, we first trained a VAE on uncorrupted CT images and developed an optimization pipeline that iteratively searches the latent space of the VAE for the point that generates an image whose forward projection best matches the measured, compromised projection data. LSR was tested on clinical data that included metal artifacts, on clinical data with simulated truncation as well as CBCT data with simulated truncation, and its performance was compared to other established artifact reduction methods.

For truncation artifacts on clinical data, LSR was benchmarked against both the classical algorithm ADT and a state‐of‐the‐art deep learning‐based detruncation method. The results demonstrated that LSR achieved artifact reduction performance comparable to the deep learning approach while outperforming the classical extension method. Additionally, we investigated the performance of LSR on CBCT data without specifically training the underlying VAE on comparable data. While the results demonstrated strong artifact suppression, certain details, such as the reconstruction of a non‐existent table resembling one from the training dataset, highlighted the need for greater diversity in the training data used for the VAE. For metal artifacts, LSR was evaluated against the NMAR algorithm. Although no artifact‐free ground truth images were available for quantitative evaluation, qualitative analysis of the corrected images revealed that LSR consistently reduced metal artifacts, underlining its robustness across different types of missing or corrupt data.

A key strength of LSR is its versatility. Unlike most artifact reduction methods, which are designed to address specific challenges, LSR is inherently generalizable. By leveraging the latent space of a VAE trained on uncorrupted data, LSR does not require retraining or fine‐tuning for each type of artifact. This suggests that LSR could potentially be applied to other scenarios such as limited‐angle reconstructions.

Despite its strengths, two limitations must be acknowledged. First, the computational efficiency of LSR is currently insufficient for time‐sensitive clinical applications. The LSR inference time is directly proportional to the number of optimization steps. In its current implementation, LSR requires 4.3 s for 1000 optimization steps on an NVIDIA GTX 3090. For a single 512×512 image, sufficient artifact reduction was achieved after approximately 500 optimization steps, with the potential for further reduction by reusing information from prior slices. Future work could focus on reducing the computational time of the LSR method.

Second, the performance of LSR is closely tied to the quality and diversity of the training data used for the VAE. In clinical practice, data heterogeneity poses a challenge, as imaging protocols, detector types, and noise levels can vary significantly. To address this, future studies could explore training the VAE on broader datasets that include data from cone‐beam CT systems, images with scatter artifacts, or data with varying noise characteristics. Alternatively, the method could be extended by incorporating a scatter model or additional noise into the forward process during the LSR update steps. This addition could enhance the overall robustness of the approach.

## CONFLICT OF INTEREST STATEMENT

The authors declare no conflicts of interest.

## Figures and Tables

**FIGURE A1:**
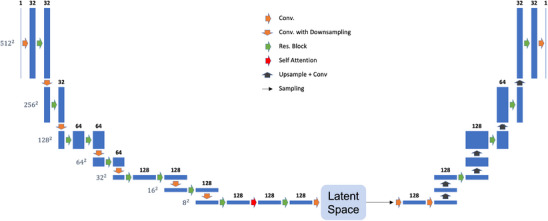
VAE architecture and training.

**FIGURE A2:**
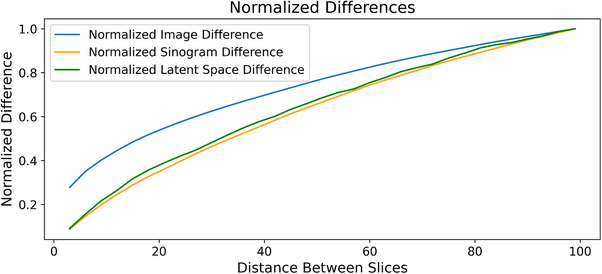
Distances of different slices in image domain (blue), sinogram domain (yellow), and encoded in latent space (green) for increasing distances in z‐direction.

**FIGURE A3:**
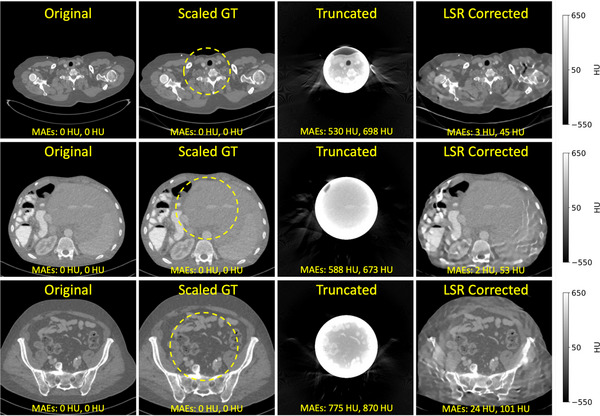
Results on simulated larger patients, generated by randomly scaling slices. Same slices as in Figure 3 shown for comparison. C=50HU,W=1200HU.

**FIGURE A4:**
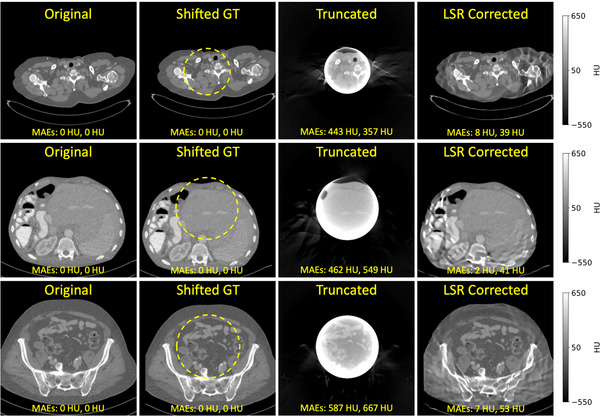
Results on simulated miscentered patients, with random shifts in x and y directions. Same slices as in Figure 3 shown for comparison. Images C=50HU,W=1200HU.

## Footnotes

1 Clinical CT measurements show little scatter due to the use of anti‐scatter grids.

## APPENDIX A

### A.1 VAE ARCHITECTURE

The backbone of our method is a VAE^34^, primarily because LSR requires a generative model with a continuous latent space that supports balanced sampling. The VAE's latent space corresponds to the parameters of a contiguous variational distribution N, so that our decoder is a well‐behaved generator function. Given a low‐dimensional latent vector z, it is able to generate a realistic representation of the object, and close neighbors of z generate neighboring objects. The inputs and outputs of the VAE are 512×512 gray scale images. The architecture of our VAE is shown in Figure A1. Each residual block sequentially applies group normalization, activation, and two convolutional layers with a kernel size of 3×3, with a residual connection that either directly adds the input or passes it through a 1×1 convolution if the input and output channels differ. The last convolutional layer in the encoder reduces the number of channels from 128 to 8, resulting in a latent space dimension of 512.

The VAE is optimized by maximizing the evidence lower bound (ELBO), with respect to the network parameters. The combined VAE hybrid loss function included L1 loss ($L_{\text{recon}}$) and Kullback–Leibler (KL) divergence loss ($L_{\text{KL}}$):

(A1)
$$\begin{array}{cc} & L=L_{\text{recon}}+w_{\text{KL}}\cdot L_{\text{KL}}\\ & L_{\text{recon}}=∥f-D\left(E\left(f\right)\right)∥\\ & L_{\text{KL}}=D_{\text{KL}}\left(N(\mu ,\sigma )∥N(0,1)\right) \end{array}$$

where ∥·∥ refers to the Euclidean norm, $w_{\text{KL}}$ to the weighting of the $L_{\text{KL}}$ term, D to the VAE decoder, E to the VAE encoder, f denotes CT images, and $D_{\text{KL}}$ is the Kullback–Leibler divergence. To optimize this architecture, we set the initial learning rate to 0.001 and the batch size to 16, training the model for 100 epochs. A learning rate scheduler with a step size of 25 epochs and a decay factor of 0.1 was employed to adjust the learning rate during training. The model was trained on a single NVIDIA GTX 3090 with 24 GB of VRAM.

### A.2 SAMPLING FROM LATENT SPACE

The paramatrization of the latent space ensures, that if the sinograms of neighboring (similar) axial slices are similar, their respective latent representations are likely to be similar as well. The latent space of the VAE is designed to capture the underlying structure and variability of the input data while mapping similar inputs to close points in the latent space.

To validate this, we conducted an experiment where we evaluated the pairwise similarity of latent vectors, sinograms, and axial slices at increasing distances. This was quantified by taking the absolute difference. The results, displayed in Figure A2, show an average over the entire test dataset.

However, the VAE generated images are generally not reproducible because, in its current implementation, the optimization problem of LSR is not convex. As a result, convergence to a global optimum cannot be guaranteed.

### A.3 ADDITIONAL EXPERIMENTS

To evaluate the performance of LSR in scenarios involving improperly centered or larger patients, we conducted additional experiments. Larger patients were simulated by scaling the slices with a random factor uniformly drawn from the interval [1.1,1.2] and displayed in Figure A3. Miscentered patients were simulated by applying a random shift to each slice in both the x and y directions, with values uniformly drawn from the intervals [−50,−25]mm and [25,50]mm and displayed in Figure A4. The results of these experiments are shown for the same patient slices as displayed in Figure 3, enabling a better comparison. A qualitative analysis over the entire test dataset is provided in Table A1. Notably, LSR shows similar performance on scaled and shifted patients within the field of measurement (FOM), and a slightly worsened performance outside the FOM.

**TABLE A1:** Performance of the LSR method for random shifts in x and y directions, with values uniformly drawn from either −50to−25 mm or 25to50 mm and scaling of the patients uniformly drawn from [1.1,1.2].

	LSR	LSR on shifted data	LSR on scaled data
$\bar{\mathrm{MAE}}$ **inside FOM** $\left[\text{HU}\right]$	15.0 ± 9.5	14.8 ± 11.2	16.4 ± 13.1
$\bar{\mathrm{MAE}}$ **outside FOM** $\left[\text{HU}\right]$	103 ± 76	127 ± 91	155 ± 83
$\bar{\mathrm{MAE}}$ **of sinogram**	0.20 ± 0.07	0.26 ± 0.16	0.29 ± 0.11

Abbreviations: FOM, field of measurement; LSR, latent space reconstruction; MAE, mean absolute error.
